# Supplementary material for: A study of the influence of sports venues on the intra-city population layout based on multi-source data—Taking Xi’an city and Zhengzhou city as examples
Source: PLoS One. 2023 May 11;18(5):e0285600. doi: 10.1371/journal.pone.0285600 (PMC10174530; doi:10.1371/journal.pone.0285600)
Supplement: Attachment — Submitted filename: REVIEWERS COMMENT_PONE-D-22-35291.docx [file pone.0285600.s001.docx]

**REVIEWER’S COMMENT**

**Journal**: PLOS ONE

**Manuscript ID**: PONE-D-22-35291

**Title**: A study of the influence of sports venues on the intra-city population layout based on multi-source data- taking Xi’an city and Zhengzhou city as examples.

**General comments**

The manuscript is well written and provides a sound understanding of the set objective it outlines in the introduction. The study examined the influence of the distribution of sport venues on the intra-city population layout in Xi’an city and Zhengzhou city. Based on spatial analysis using MGWR, GWR and linear fitting, the study found that the spatial distribution of sport facilities venue is a good indicator of population distribution. While the manuscript is good, it can be further improved and its chances of being published greatly increased if the following specific comments are considered and the manuscript duly revised.

**Specific Comments**

i. *Topic*

I suggest the topic is modified to read: *“Influence of sports venues on the intra-city population layout based on multi-source data. A case of Xi’an city and Zhengzhou city”*

ii. *Abstract*

It will be helpful if the abstract includes a couple of recommendations based on the findings of the study as stated in the abstract section. Furthermore line 9 should be checked for a repetition of the word *‘sports’*.

iii. *Introduction*

The introduction is well written.

iv. *Materials and methods*

It will be good if authors could respond to the following issues and make them clearer in the materials and methods section of the manuscript:

a) Kindly clarify the paragraph (line 104 to 108) in terms of the two sets of data used for the study. Why was there a need for the second dataset (data from the Public Information website)
b) There should be consistency in the use of ‘inner city’ or ‘intra-city’ which seems to be used interchangeably in the manuscript.
c) Line 128, how do you convert .tiff data to vector and extract the built-up area? Was there no image classification done? An explanation of the process will do.
d) At line 146, I would suggest you use the caption: data analysis.
e) Which analytical software was used in this study?
f) In the introduction and abstract, you mention the use of OLS and Linear fitting as part of the analytical tool used in your study, however, it has not been explained in your data analysis section.
v. *Results*
a) Unfortunately, authors failed to mention that they run correlation analysis in their write up at the data analysis section even though this is mentioned and discussed at the results section.
b) Again, the scatter plots show a normal relationship than a correlation analysis. If it was a correlation analysis, kindly present the significance level.
c) It will be useful to explain the OLS results in the data analysis section as presented in Table.
