# Supplementary material for: A study of the influence of sports venues on the intra-city population layout based on multi-source data—Taking Xi’an city and Zhengzhou city as examples
Source: PLoS One. 2023 May 11;18(5):e0285600. doi: 10.1371/journal.pone.0285600 (PMC10174530; doi:10.1371/journal.pone.0285600)
Supplement: Attachment — Submitted filename: re-two.docx [file pone.0285600.s002.docx]

Response to Reviewer 2 Comments

Dear PLOS ONE Editorial Board Editors and Reviewers.

Hello!

Thank you very much for your review of "Influence of sports venues on the intra-city population layout based on multi-source data. A case of Xi'an city and Zhengzhou city". We have addressed the reviewer's questions. We have carefully answered the reviewer's questions and made detailed revisions to the paper according to the reviewer's comments, and the revised parts are marked in red with revision status. The answers to the questions raised by the reviewers are as follows.

i. *Topic*

**Point 1:** I suggest the topic is modified to read: *“Influence of sports venues on the intra-city population layout based on multi-source data. A case of Xi’an city and Zhengzhou city”*

**Response 1:** Thank you for your suggestions, we have revised the article in response to your suggestions.

ii. *Abstract*

**Point 1:** It will be helpful if the abstract includes a couple of recommendations based on the findings of the study as stated in the abstract section. Furthermore line 9 should be checked for a repetition of the word *‘sports’*.

**Response 1:** Thank you for your careful reading of the study abstract, which begins with the last sentence of the abstract presenting: The study recommends that the layout of sports venues should be planned in a targeted and scientific manner, controlling the increase of sports venues in areas with special functions such as administration, business, tourism and education, while planning more sports venues in other areas appropriately due to needs, and reasonably promoting the sustainable and high-quality development of different areas; After checking the ninth line there is a duplicate of "sports", and the duplicate word was deleted.

iii. *Introduction*

**Point 1:** The introduction is well written.

**Response 1:** Thank you for your recognition and compliments!

iv. *Materials and methods*

It will be good if authors could respond to the following issues and make them clearer in the materials and methods section of the manuscript:

**Point 1:** Kindly clarify the paragraph (line 104 to 108) in terms of the two sets of data used for the study. Why was there a need for the second dataset (data from the Public Information website)

**Response 1:** Thank you for your question, first of all, POI data is a kind of emerging open data, in order to improve the scientific nature of the study, we used the more reliable Sports Authority website to verify the data; at the same time, we re-examined the original manuscript and deleted the inappropriate words, and now the paragraph is as follows: The POI data for sports venues in Xi'an and Zhengzhou in the study were obtained from Baidu Map. These data include basic information such as the name, address, and geographic coordinates. After data cleaning, the number of sports venues in the two cities was obtained by combining the public information from the websites of the Xi'an and Zhengzhou Sports Bureaus: 12,316 and 14,972, respectively.

**Point 2:** There should be consistency in the use of ‘inner city’ or ‘intra-city’ which seems to be used interchangeably in the manuscript.

**Response 2:** We have carefully read the original manuscript and standardized its language, using the term "intra-city".

**Point 3:** Line 128, how do you convert .tiff data to vector and extract the built-up area? Was there no image classification done? An explanation of the process will do.

**Response 3:** The process of converting .tiff data into vector data is tedious and not the focus of this paper, so the process is explained as follows: using Landsat TM/ETM/OLI remote sensing images as the main data source, after image fusion, geometric correction, image enhancement and stitching, the national land use types are classified into 6 primary classes, 25 secondary classes and some tertiary classes by human-computer interaction visual interpretation. The land use data are classified into 6 primary categories, 25 secondary categories and some tertiary categories.

**Point 4:** At line 146, I would suggest you use the caption: data analysis.

**Response 4:** We have changed "Research Methodology" to "Data Analysis" in response to your suggestion.

**Point 5:** Which analytical software was used in this study?

**Response 5:** In this study, ENVI5.3 and ArcGIS10.6 were used in the data pre-processing stage, ArcGIS10.6 was used for the kernel density estimation method, and MGWR2.2 was used for the multi-scale geo-weighted regression.

**Point 6:** In the introduction and abstract, you mention the use of OLS and Linear fitting as part of the analytical tool used in your study, however, it has not been explained in your data analysis section.

**Response 6:** In this study, OLS, GWR and MGWR were used in doing regression analysis to obtain their regression parameters R2, AICc and residual sum of squares respectively and to compare the obtained parameters, it was concluded that MGWR was more suitable for this study, so OLS, GWR and MGWR were used, while only the results of MGWR were needed in the analysis part.

v. *Results*

**Point 1:** Unfortunately, authors failed to mention that they run correlation analysis in their write up at the data analysis section even though this is mentioned and discussed at the results section.

**Response 1:** Thank you for raising this issue and we have added a correlation analysis to the data analysis section as follows:

3.3.2. Pearson Correlation Analysis

Pearson correlation refers to the simple linear correlation between random variables X and Y whose joint distribution obeys a two-dimensional normal distribution. The correlation between X and Y is represented by the simple correlation coefficient r. The Pearson correlation coefficient is used to measure whether the two data sets are on a straight line and is calculated as follows [33]:

| $r_{xy}=\frac{n\sum x_{i}y_{i}-\sum x_{i}\sum y_{i}}{\sqrt{n\sum x_{i}^{2}-(\sum x_{i})^{2}\sqrt{n\sum y_{i}^{2}-(\sum y_{i})^{2}}}}$ | (5) |
| --- | --- |

The value of Pearson correlation coefficient ranges from [-1, 1], when r=0, it means there is no linear relationship between the two; when r>0, there is a certain linear positive relationship between the two, which means X has a certain positive relationship to Y, and the opposite is negative.

**Point 2:** Again, the scatter plots show a normal relationship than a correlation analysis. If it was a correlation analysis, kindly present the significance level.

**Response 2:** To represent the results of correlation analysis more clearly, we added the results of Pearson correlation analysis in 4.3.1 and changed Figure 6 to add the correlation coefficient heat map.

4.3.1. Overall Correlation Analysis

Sports and leisure activities that enrich the spare time of the general public can represent people's aspiration for a better life and are closely related to the development of cities. Therefore, as a carrier of residents' leisure and sports activities, sports venues not only have the function of leisure and entertainment but also have certain social symbolic functions. Additionally, studies have proven that the distribution of sports venues is closely related to the size of the population [24,28,29]. To explore the influence of the density of the stadium distribution on the intra-city population distribution in Xi'an and Zhengzhou cities, scatter plots and Pearson correlation analysis were created based on normalized data from Xi'an and Zhengzhou cities using Stata 16 and Origin 2018 software (Figure 6).

**Figure 6.**Heat map of linear fit and correlation coefficient between the intensity of sports stadium distribution and population density.

As shown in Figure 6, the models of Xi'an and Zhengzhou cities fit better based on the overall perspective, and the slopes of the fitted lines are positive. The coefficients obtained from the Pearson correlation analysis were all positive and significant at the 0.001 level. This means that the distribution of sports venues in both Xi'an and Zhengzhou cities can promote the growth of the intra-city population at the global scale. The slope of the fitted line and the correlation coefficient are higher in Zhengzhou, which means that the intra-city population in Zhengzhou is more sensitive to the distribution of sports venues.

**Point 3:** It will be useful to explain the OLS results in the data analysis section as presented in Table.

**Response 3:** After the comparison of the three regression methods, it was found that the r2 of OLS was extremely small and the value of AICc was too large, and the regression effect was not satisfactory. The authors, after all the discussions, concluded that the results obtained from the OLS regression model could not better explain the influence of stadium distribution on population layout.

Yours sincerely,

Yang Liu
